# Outcomes of Primary vs. Delayed Strategy of Implanting a Cardiac Monitor for Unexplained Syncope

**DOI:** 10.3390/jcm11071819

**Published:** 2022-03-25

**Authors:** Ekrem Yasa, Theodoros Intzilakis, Fabrizio Ricci, Olle Melander, Viktor Hamrefors, Richard Sutton, Artur Fedorowski

**Affiliations:** 1 Department of Clinical Sciences, Lund University, 214 28 Malmö, Sweden; ekrem.yasa@med.lu.se (E.Y.); theodoros.intzilakis@skane.se (T.I.); fabrizio.ricci@unich.it (F.R.); olle.melander@med.lu.se (O.M.); viktor.hamrefors@med.lu.se (V.H.); r.sutton@imperial.ac.uk (R.S.); 2 Department of Cardiology, Skåne University Hospital, 214 28 Malmö, Sweden; 3 Department of Neuroscience, Imaging and Clinical Sciences, “G. d’Annunzio” University of Chieti-Pescara, 66100 Chieti, Italy; 4 Department of Internal Medicine, Skåne University Hospital, 214 28 Malmö, Sweden; 5 National Heart and Lung Institute, Hammersmith Hospital Campus, Imperial College, London W12 0HS, UK; 6 Department of Cardiology, Karolinska University Hospital, 171 64 Solna, Sweden; 7 Department of Medicine, Karolinska Institute, 171 77 Stockholm, Sweden

**Keywords:** cardiac arrhythmias, electrocardiographic monitoring, implantable loop recorder, pacemaker, syncope, orthostatic hypotension, autonomic nervous system, cardiovascular autonomic testing

## Abstract

Objective: Implantable cardiac monitors (ILR) have an important role in diagnosing unexplained syncope. However, outcomes of primary vs. delayed ILR implantation after initial syncope evaluation have not been explored. Methods: A total of 1705 patients with unexplained syncope were prospectively enrolled in the SYSTEMA (Syncope Study of Unselected Population in Malmö) cohort. Patients who underwent cardiovascular autonomic testing (CAT) and ILR were grouped into those referred to CAT after ILR implantation (primary ILR) and those in whom ILR was indicated after CAT (post-CAT ILR). Results: One-hundred-and-fifteen patients (6.7%) received ILRs. ILR recipients were older (58 vs. 52 years; *p* = 0.002), had more syncope recurrences (6 vs. 4; *p* < 0.001), more traumatic falls (72% vs. 53%; *p* < 0.001), and less prodrome (40% vs. 55%; *p* = 0.005) than patients without ILRs. During follow-up ≥16 months after ILR, 67 (58%) had normal sinus rhythm, 10 (8.7%) had sinus arrest, 10 (8.7%) AV-block, 13 (11.3%) atrial fibrillation, 9 (7.8%) supraventricular tachycardia, 4 (3.5%) sinus tachycardia and 2 (1.7%) ventricular tachycardia with clinical symptom reproduction. There were 52 patients (45%) in the primary-ILR group and 63 (55%) in the post-CAT ILR group. Proportions of negative ILR monitoring (17/52 vs. 25/63; *p* = 0.56) and pacemaker implantations (7/52 vs. 15/63; *p* = 0.23) did not differ between groups. Baseline ECG conduction disorders predicted pacemaker implantation (*n* = 11/17; odds ratio:10.6; 95%CI: 3.15–35.3; *p* < 0.001). CAT was more often positive (73% vs. 40%; *p* < 0.001) in primary-ILR group. Conclusions: Primary ILR implantation was associated with more positive CAT compared with delayed ILR implantation, but negative monitoring and pacemaker implantations were not different between groups. ECG conduction disorders predicted subsequent pacemaker implantation.

## 1. Introduction

Syncope is a common clinical symptom defined as transient loss of consciousness (T-LOC) due to cerebral hypoperfusion, with a rapid onset, short duration and total recovery [1]. The current European Society of Cardiology (ESC) syncope guidelines state that when syncope is unexplained, a stepwise algorithm should be followed with cardiovascular autonomic testing (CAT) and prolonged ECG monitoring with implantable loop recorder (ILR), as main diagnostic components [1]. However, the Guidelines are not prescriptive as to which investigation should take precedence. The role of ILRs in the diagnosis of unexplained syncope is not yet completely established, as according to guidelines, when cardiac arrhythmic syncope likelihood is high, primary ILR implantation should be considered, i.e., prior to CAT [1]. However, the outcomes of ILR in relation to the timing of CAT have not been well investigated. This study aimed to explore the results of primary vs. post-CAT ILR monitoring in a tertiary center setting offering full-scale syncope workup. In addition, we planned to identify ILR outcome predictors from baseline parameters.

## 2. Materials and Methods

### 2.1. Study Settings

The single-centre prospective Syncope Study of Unselected Population in Malmö (SYSTEMA) was established to investigate systematically and manage patients with unexplained syncope [2]. Between September 2008 and November 2016, 1705 syncope patients were enrolled and investigated in the tertiary Syncope Unit of Skåne University Hospital in Malmö, Sweden. During the investigation, participants underwent cardiovascular autonomic assessment, including carotid sinus massage (CSM), active stand test, head-up tilt (HUT), and ILR implantation, if the etiology of syncope was not established. Additional tests, such as echocardiography, Holter monitoring, exercise ECG, coronary angiography, brain imaging, carotid duplex ultrasonography and EEG were carried out prior to or in parallel with the main syncope workup if deemed appropriate by the referring physician. Some of these referred patients had an ILR implanted prior to referral; the reasons for referral of these patients were either negative or inconclusive ILR data, e.g., syncope recurrence without diagnostic ECG changes on ILR. These patients were included in the standard CAT workup in the Syncope Unit and followed as other ILR patients.

### 2.2. Study Population

Inclusion criteria: consecutive patients with unexplained syncope who underwent both ILR implantation and CAT were included in the current nonrandomized study.

The “primary-ILR” subgroup included patients who had ILRs implanted, with the initial indication simply of unexplained syncope, prior to referral and with either negative or inconclusive ILR data in relation to syncope prior to CAT. The “Post-CAT ILR” subgroup included patients in whom ILR was implanted after CAT when CAT results were negative or judged unable to explain the clinical symptom.

The study complied with the Declaration of Helsinki, the Regional Ethical Review Board in Lund, Sweden accepted the study protocol (ref no 82/2008), and all study participants gave their written informed consent.

### 2.3. Patients and Public Involvement

The patients and public were not involved in the designing of, recruitment to, or conduct of the present study.

### 2.4. Examination Protocol

Cardiovascular autonomic tests (CAT) included supine and upright CSM according to the Newcastle protocol [3], active standing [1] and HUT testing at 60–70° plus optional nitroglycerin provocation according to the Italian protocol [4]. The patients were instructed to take their regular medication and fast for 2 h before testing, and they were allowed to drink water freely. ECG and blood pressure were continuously monitored using a non-invasive method (Nexfin monitor, BMEYE, Amsterdam, the Netherlands, or Finapres Nova, Enschede, The Netherlands) and subsequently analysed offline using a dedicated program provided by the monitor manufacturer. Furthermore, patients were asked to complete a questionnaire including medical history, frequency, duration, and features of syncope-related symptoms.

### 2.5. Implantable Loop Recorders

ILRs were implanted according to the manufacturer’s instructions, and monitoring was performed by a dedicated unit run by specialized pacemaker nurses at the study location or collaborating centers. One-hundred-twelve Reveal^TM^ devices of first and second-generation (Medtronic Inc, Minneapolis, MN, USA) and three Confirm^TM^ devices (Abbott SJM, Saint Paul, MN, USA) were implanted.

### 2.6. Clinical Follow-Up

Medical records of all ILR patients were analyzed by the first and senior author (EY and AF) for adjudication. The following data were retrieved: date of inclusion in the study, ECG rhythm after index syncopal event retrieved from medical records; date of ILR implantation, syncope or symptom recurrences during ILR follow-up; date and details of ILR-derived diagnosis, if valid; date of pacemaker implantation, if performed; number and type of ancillary tests performed during initial syncope workup; and results of cardiovascular autonomic testing. Patients were followed-up until 28 February 2019.

### 2.7. Statistical Analysis

First, all patients with ILR were compared with those patients who did not receive ILR; next, patients in the primary-ILR subgroup were compared with the post-CAT-ILR subgroup. Syncope groups were compared using appropriate statistical tests: ANOVA or Mann-Whitney U-test for continuous variables, and Pearson’s Chi-square or Fisher’s exact test, if appropriate, for categorical variables. Baseline data, including syncope characteristics and past medical history, were analyzed. Likewise, all tests performed during syncope workup, results of CAT and ILR monitoring outcomes were compared. Predictors of pacemaker implantation were analyzed using logistic regression in univariable and multivariable-adjusted (age and sex) models. Data were analyzed using IBM SPSS software version 27 (Armonk, New York, NY, USA) and GraphPad Prism V.6.00, GraphPad Software (La Jolla, CA, USA), www.graphpad.com (accessed on 5 February 2022). A *p*-value of <0.05 was considered statistically significant. The data underlying this article will be shared at reasonable request to the corresponding author.

## 3. Results

Of the 1705 patients, a total of 115 patients (6.7%) received ILRs. The 115 patients with ILR were older (58 years vs. 52 years; *p* = 0.002), had more syncope episodes (6 vs. 4; *p* < 0.001), more fall trauma (72% vs. 53%; *p* < 0.001), and less prodrome (nausea, cold sweat; 40% vs. 55%; *p* = 0.005) than the remaining 1590 patients who did not receive ILRs. Other syncope-associated symptoms, i.e., palpitations, supine syncope, and dizziness while standing, did not differ between patients with and without ILRs, as neither conducted a history of cardiovascular disease (hypertension, ischaemic heart disease, stroke, heart failure, atrial fibrillation) and diabetes (*p* > 0.05; data not shown). ILR-patients were more often hospitalized due to syncope (59% vs. 43%; *p* = 0.001) than those who did not receive ILRs.

Of 115 ILR recipients, 52 (45%) had received ILRs prior to referral and CAT (primary-ILR group), and 63 (55%) after an inconclusive CAT-based diagnostic workup for syncope (post-CAT ILR group) (Table S1). Patients with primary vs. post-CAT ILR did not significantly differ in age (60 vs. 57 years; *p* = 0.412) and were numerically more likely men (73% vs. 56%; *p* = 0.056). Other clinical parameters (recurrent syncope, prodromes before syncope, fall trauma related to syncope, palpitations, supine syncope) and comorbidities (hypertension, heart failure, stroke, atrial fibrillation, diabetes) did not differ between the groups (Table 1). Primary-ILR patients were more extensively investigated prior to ILR-implantation compared with post-CAT group: they were more often hospitalized (70% vs. 51%; *p* = 0.039), and were more often examined by brain CT/MRI (90% vs. 70%; *p* = 0.008), echocardiography (92% vs. 73%; *p* = 0.009), Holter-ECG (88% vs. 67%; *p* = 0.007), and EEG (68% vs. 40%; *p* = 0.003). Figure S1 describes the time relationship between ILR and CAT in the study population.

**Figure 1:**
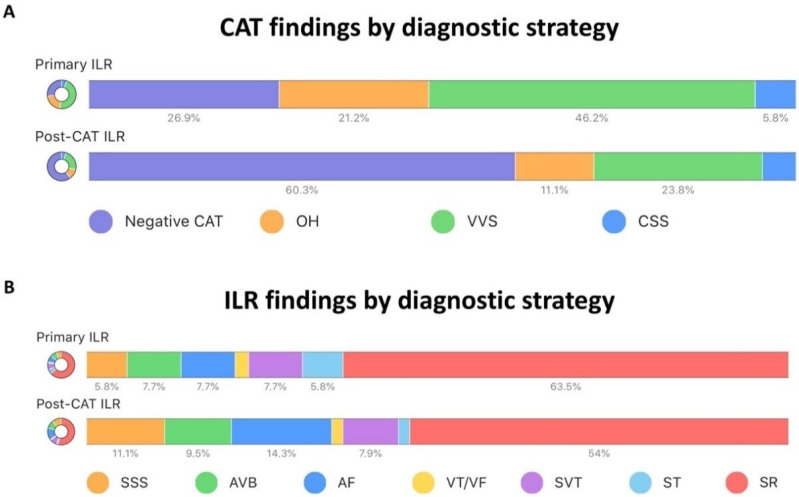
Unexplained syncope patients are compared in terms of diagnostic findings in the two groups: primary ILR implantation and post-CAT ILR implantation. Panel (**A**): CAT findings by diagnostic strategy. Panel (**B**): ILR findings by diagnostic strategy. CAT, cardiovascular autonomic testing; ILR, implanted loop recorder. VVS, vasovagal syncope; OH, orthostatic hypotension; CSS, carotid sinus syndrome; SSS, sick sinus syndrome; AVB, atrioventricular block; AF, atrial fibrillation; VT/VF, ventricular tachycardia/ventricular fibrillation; SVT, supraventricular tachycardia; ST, sinus tachycardia; SR, sinus rhythm.

**Figure 2:**
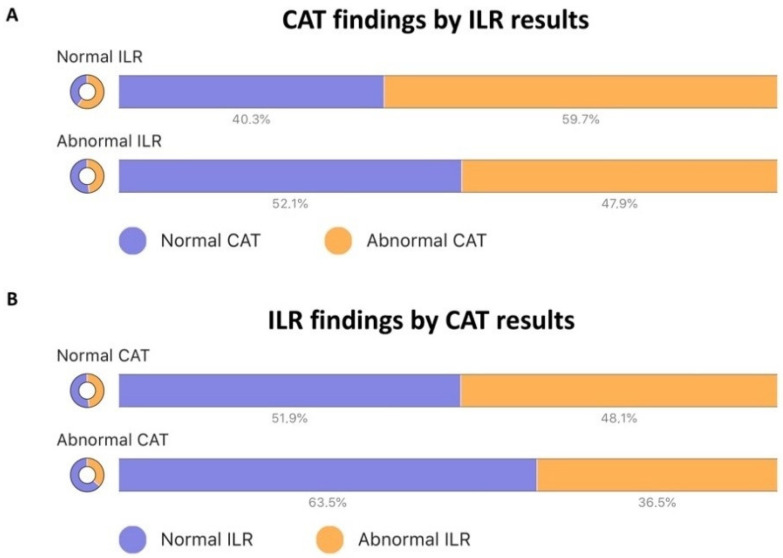
Distribution of normal and abnormal ILR and CAT findings in patients with unexplained syncope. Panel (**A**): CAT findings by ILR results. Panel (**B**): ILR findings by CAT results. CAT, cardiovascular autonomic testing; ILR, implanted loop recorder.

During follow-up, for at least 16 months after ILR implantation, 67 patients (58%) had normal sinus rhythm, whereas 10 (8.7%) had sinus arrest, 10 (8.7%) AV block, 13 (11.3%) atrial fibrillation, nine (7.8%) supraventricular tachycardia, four (3.5%) sinus tachycardia, and two (1.7%) ventricular tachycardia (with symptom reproduction) detected by ILR. The main findings of CAT and ILR monitoring for each group are reported in Table 2, Figure 1 and Figure 2 according to the previously proposed International Study of Syncope of Unexplained Etiology (ISSUE) classification with modifications [5].

Briefly, sick sinus syndrome and atrial fibrillation were more frequently found in the post-CAT ILR group, whereas sinus tachycardia was more frequent in the primary-ILR group. Normal sinus rhythm was observed in the same proportion in both groups (primary ILR group, 63%, and post-test ILR group, 54%). While being monitored by ILR, 46 patients experienced syncope recurrence, 26 in the primary-ILR and 20 in the post-CAT ILR group (*p* = 0.057 for intergroup comparison). Negative ILR monitoring, defined as no syncope recurrence and no diagnostic ECG findings did not differ between the groups (17/52 vs. 25/63; *p* = 0.56). During syncope recurrence, sinus rhythm was more often observed in the primary-ILR group (16/52 vs. 9/63; *p* = 0.042). There were 22 pacemakers implanted: 20 for sinus arrest or AV block, one due to slow atrial fibrillation, plus one ICD implant for VT. Of these, seven were implanted in the primary-ILR group, and 15 in the post-CAT-ILR group (*p* = 0.23). The second detected VT patient received pharmacological treatment as per physician decision. Other diagnostic findings, such as de novo atrial fibrillation and supraventricular tachycardia resulted in drug optimization and/or referral for invasive electrophysiology.

In univariable logistic regression analysis, age (odds ratio (OR) per year: 1.05; 1.01–1.09; *p* = 0.008), higher systolic blood pressure (OR per 1 mmHg: 1.03; 1.01–1.05; *p* = 0.008), and conduction disorder on ECG at initial evaluation (11 implantations among 17 patients with AV block—any degree, LBBB, or RBBB; OR, 14.5, 4.47–47.0; *p* < 0.001) predicted pacemaker implantation. In a multivariable-adjusted model, the only independent pacemaker implantation predictor was conduction disorder (OR 10.6; 3.15–35.3; *p* < 0.001).

Other clinical parameters, such as the number of syncope episodes, the occurrence of prodrome before syncope, palpitation, fall trauma, history of cardiovascular disease and diabetes, were not predictive of pacemaker implantation (data not shown).

Results of CAT stratified by ILR implanting strategy are presented in Table 3 and Figure 2. Patients with ILR implanted before CAT had more positive tests (73% vs. 40%; *p* < 0.001) and more frequent diagnoses of VVS and OH, compared with patients who received ILR after testing, meaning that cardiovascular autonomic dysfunction was more frequently diagnosed in patients with an ILR implanted before referral.

**Figure 3:**
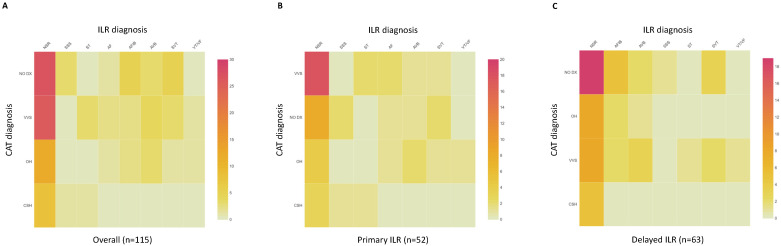
Heat map showing frequency of diagnosis by ILR and CAT: (**A**) overall population; (**B**) primary ILR subgroup; (**C**) post-CAT (delayed) ILR subgroup. AF, atrial fibrillation; AVB, atrioventricular block; CAT, cardiovascular autonomic testing; CSS, carotid sinus syndrome; ILR, implanted loop recorder; NSR, normal sinus rhythm; OH, orthostatic hypotension; SSS, sick sinus syndrome; ST, sinus tachycardia; SVT, supraventricular tachycardia; VT/VF, ventricular tachycardia/ventricular fibrillation; VVS, vasovagal syncope.

As shown in Figure 2A, the proportions of abnormal CAT results were not significantly different between the groups of normal vs. abnormal ILR monitoring results (48% vs. 60%; *p* = 0.21). Similarly, when ILR outcomes were stratified by normal vs. abnormal CAT results (Figure 2B), the frequency of abnormal ILR findings did not differ between the groups (48% vs. 36%; *p* = 0.21). Heat maps (Figure 3) present the individual results for each of 115 patients with ILR stratified by implantation strategy (primary vs. post-CAT).

## 4. Discussion

This single-centre prospective study compared diagnostic yield and therapeutic implications of a primary ECG loop recorder implantation strategy versus comprehensive cardiovascular autonomic testing (head-up tilt, active standing, and carotid sinus massage) and subsequent ECG loop recorder implantation in a population of unexplained syncope patients. Our data demonstrate a nonsignificant difference in the number of final diagnoses achieved and the proportion of pacemaker implantations between the two strategies. However, as expected, the primary ILR implantation strategy resulted in a higher proportion of positive findings during CAT, although the primary ILR group was more extensively examined, prior to referral, with multiple investigations, such as echocardiography, Holter ECG and brain imaging compared with those examined first with CAT.

These are important observations because these two diagnostic strategies, ILR and CAT, both appear as reasonable clinical options, in line with current syncope guidelines. However, there are other considerations than simply a choice between early ILR implantation, with possible additional CAT, and CAT with ILRs selected only when CAT yields no definite diagnosis.

Assessment of cardiovascular autonomic function and reflex syncope susceptibility gives several distinct patient management advantages:

- confirmation of diagnosis by reproduction of spontaneous symptoms of VVS on tilt [6]
- patient education about prodromes and counter-pressure maneuvers on tilt in VVS [6]
- a basis for pacemaker selection in VVS [7],
- prognostic information with respect to future syncope recurrence, especially in the context of pacing therapy [8]
- a diagnosis of carotid sinus syndrome which may call for a different pacemaker program [1],
- understanding the role of the vasodepressor component in both CSS and VVS implying a possible reduction in hypotensive medication or even addition of medication to support blood pressure [9],
- diagnosis of OH by active standing and delayed OH by tilt [1,10,11].

Thus, the two approaches must be considered complementary, as supported by the comparison displayed in Figure 2. Neither positive nor negative CAT results predicted ILR outcome, which suggests that CAT identifies potential syncope mechanisms that a) may exist in parallel to arrhythmic mechanisms but are not responsible for the syncope under investigation; b) two or more mechanisms may exist in parallel and equally contribute to syncope. A similar observation was made regarding patients with positive vs. negative ILR monitoring: in both groups, the proportion of positive CAT results was not different. This illustrates the complexity of unexplained syncope investigation: some patients had positive CAT only, some had positive ILR monitoring only, some had both, and a group of patients had neither positive CAT nor ILR, which is where the current challenge of syncope management lies. Consequently, a simple ILR strategy alone is inadequate, while a dual diagnostic strategy, CAT and ILR, offers the best available patient management. This method of investigation is fully compatible with ESC guidelines [1].

Our data also support the selection of ILR after CAT as there will be cost savings in less ILR use. Based on epidemiological data, around 70% of syncope etiologies may be captured by CAT, whereas around 15% by long-term ECG monitoring. If the pre-test probability is very high for the latter, for instance in chronic conduction disorders and history of syncope suggesting non-orthostatic sudden-onset scenario, a primary-ILR strategy should be preferable, as the probability of recurrent arrhythmia in the post-syncopal period is high. For the remaining patients, a CAT-first strategy would be preferential and cost-effective. An exception to the dual approach could be made when 12-lead ECG shows evidence of conduction disorders, especially LBBB. Unsurprisingly, we found that these abnormalities were powerful predictors of the need for pacing. Recently, a meta-analysis has shown that ILR is a superior approach to both diagnostic electrophysiological study and immediate pacemaker implantation [12]. The issue of recurrent syncope after pacemaker implantation in patients for whom syncope was the main indication for pacing therapy has recently come into focus [13,14,15]. The report of Palmisano et al. in 2020 [13] demonstrated the importance of autonomic status assessment before implantation to predict the likelihood of syncope recurrence after pacing, a subject also covered by the 2018 ESC guidelines [1]. Further supporting this, in the same cohort as in the current study, the most common causes of syncope recurrence in paced patients were orthostatic hypotension and vasovagal syncope [16], which can be diagnosed by CAT. This further emphasizes the use of the dual strategy in which CAT is preferably done prior to ILR implantation unless there are specific signs, such as conduction abnormalities or high-risk settings suggesting cardiac arrhythmia supporting a primary ILR implantation.

The yield of arrhythmia diagnosis with ILRs is clearly superior to CAT, which is supported by our data and, again, fully assimilated in guidelines [1].

Interestingly, in the SPRITELY (Syncope: Pacing or Recording in the Later Years) pragmatic randomized trial enrolling patients ≥50 years of age with a bifascicular block, a strategy of empiric permanent pacing failed to reduce syncope recurrence compared with an ILR-guided strategy, further confirming that a substantial likelihood of syncope recurrence in patients who receive a permanent pacemaker is likely caused by vasodepressor syncope [17].

### Limitations

We acknowledge a few limitations that must be addressed. Firstly, this is a small single-centre prospective study that has intrinsic limitations and obvious selection bias. Secondly, patients were not prospectively randomized to ILR as an initial strategy or CAT followed by ILR. Consequently, despite sharing many similar characteristics, the two populations cannot be held completely comparable. However, our findings fully support the idea of performing a randomized clinical trial with appropriate patient selection.

Finally, longer-term monitoring could have increased the diagnostic yield of ILR; indeed, when a strategy of prolonging monitoring is chosen, monitoring should be maintained for several years until a diagnosis is established [18].

## 5. Conclusions

A minority of patients with unexplained syncope require monitoring with an implantable loop recorder. While early-ILR and CAT-first strategies are widely practiced, primary the CAT strategy offers a valuable and cost-effective approach in patient management, unlocking diagnoses of vasovagal syncope, orthostatic hypotension, and carotid sinus syndrome, and recurrent syncope prediction after pacing. The yield of ILR monitoring is a cardiac arrhythmia in almost 50% of patients, sick sinus syndrome/sinus arrest being the most frequent event, even in a relatively short monitoring period. Around 20% of monitored patients will receive a pacemaker, strongly predicted by the presence of conduction disorders on resting ECG.

## Figures and Tables

**Table 1:** Baseline characteristics by diagnostic strategy.

	Total (*n* = 115)	Primary ILR (*n* = 52)	Post-CAT ILR (*n* = 63)	*p*-Value
Age, years	58 ± 18	60 ± 17	57 ± 20	0.412
Male sex, *n* (%)	73 (63)	38 (73)	35 (56)	0.052
Hypertension, *n* (%)	38 (33)	16 (31)	22 (35)	0.689
GFR, mL/min	95 ± 34	95 ± 32	95 ± 36	0.998
Current smoking, *n* (%)	19 (16)	10 (19)	9 (14)	0.477
Previous CAD, *n* (%)	13 (11)	2 (4)	11 (17)	0.024
Previous stroke, *n* (%)	7 (6)	3 (6)	4 (6)	0.939
Cancer, *n* (%)	11 (10)	8 (16)	3 (5)	0.045
Diabetes, *n* (%)	8 (7)	6 (11)	2 (3)	0.079
Heart failure, *n* (%)	3 (3)	1 (2)	2 (3)	0.700
Atrial fibrillation, *n* (%)	11 (10)	7 (14)	4 (6)	0.173
Beta-blocker, *n* (%)	23 (20)	10 (20)	13 (21)	0.892
Diuretics, *n* (%)	15 (13)	6 (11)	9 (14)	0.663
RAAS inihibitors, *n* (%)	27 (23)	11 (21)	16 (25)	0.593
Nitrates, *n* (%)	2 (2)	1 (2)	1 (2)	0.891
SSRI, *n* (%)	14 (12)	6 (11)	8 (13)	0.850
Prodrome, *n* (%)	41 (36)	19 (37)	22 (35)	0.796
Fall trauma, *n* (%)	82 (72)	37 (72)	45 (71)	0.895
Palpitations, *n* (%)	27 (24)	14 (27)	13 (21)	0.395
Supine syncope, *n* (%)	24 (21)	11 (22)	13 (21)	0.903

CAD, coronary artery disease; CAT, cardiovascular autonomic testing; GFR, glomerular filtration rate; ILR, implantable loop recorder; RAAS, renin angiotensin aldosterone system; SSRI, selective serotonin reuptake inhibitors.

**Table 2:** ILR findings in patients implanted before and after CAT (46 patients experienced syncope during the monitoring period).

ILR Finding	Total (*n* = 115)	Issue	Primary ILR (*n* = 52)	Post-CAT ILR (*n* = 63)
Sinus arrest or sinus bradycardia <40 bpm	10	1A/2	3	7
AV block	10	1C	4	6
Normal sinus rhythm	67	3	33	34
Sinus tachycardia	4	4A	3	1
Atrial fibrillation	13	4B	4	9
SVT	9	4C	4	5
Ventricular tachycardia	2	4D	1	1
Pacemaker implantation	22		7	15 *
Syncope without any of the above arrhythmias (normal sinus rhythm at syncope)	25	3	16	9
Negative (no arrhythmia and no syncope)	42	3	17	25

CAT, cardiovascular autonomic testing; ILR, implantable loop recorder; ISSUE, International Study of Syncope of Unknown Etiology Classification; AV, atrioventricular; SVT, supraventricular tachycardia; * including one implantable cardioverter-defibrillator and one VVIR pacemaker due to slow atrial fibrillation in post-test ILR group.

**Table 3:** Cardiovascular autonomic test (CAT) findings in patients with implantable loop recorder received before and after CAT.

CAT Finding	Total (115 pts)	Primary ILR (52 pts)	Post-CAT ILR (63 pts)	*p*-Value
VVS	39 (34%)	24 (46%)	15 (24%)	0.035
OH	18 (16%)	11 (21%)	7 (11%)	0.058
CSS	6 (5%)	3 (6%)	3 (5%)	0.26
Any positive result	63 (55%)	38 (73%)	25 (40%)	<0.001
All negative tests	52 (45%)	14 (27%)	38 (60%)	<0.001

VVS, vasovagal syncope; OH, orthostatic hypotension; CSS, carotid sinus syndrome.

## Data Availability

The authors agree to make data and materials supporting the results or analyses presented in their paper available upon reasonable request.

## Supplementary Materials

The following supporting information can be downloaded at: https://www.mdpi.com/article/10.3390/jcm11071819/s1, Figure S1. Median time interval between cardiovascular autonomic testing (CAT) and implantable loop recorder (ILR) in primary ILR and post-CAT (delayed) ILR subgroups in the SYSTEMA cohort; Table S1: Individual patient diagnosis by post-CAT or primary ILR strategy.
