# Supplementary material for: Outcomes of Primary vs. Delayed Strategy of Implanting a Cardiac Monitor for Unexplained Syncope
Source: J Clin Med. 2022 Mar 25;11(7):1819. doi: 10.3390/jcm11071819 (PMC8999882; doi:10.3390/jcm11071819)
Supplement: Supplementary file 1 [file jcm-11-01819-s001.zip › jcm-1607012-supplementary.pdf]

Outcome of primary vs delayed strategy of implanting a cardiac monitor for unexplained syncope

Online Supplementary Material

Figure S1. Median time interval between cardiovascular autonomic testing (CAT) and implantable loop recorder (ILR) in primary ILR and post-CAT (delayed) ILR subgroups in the SYSTEMA cohort.

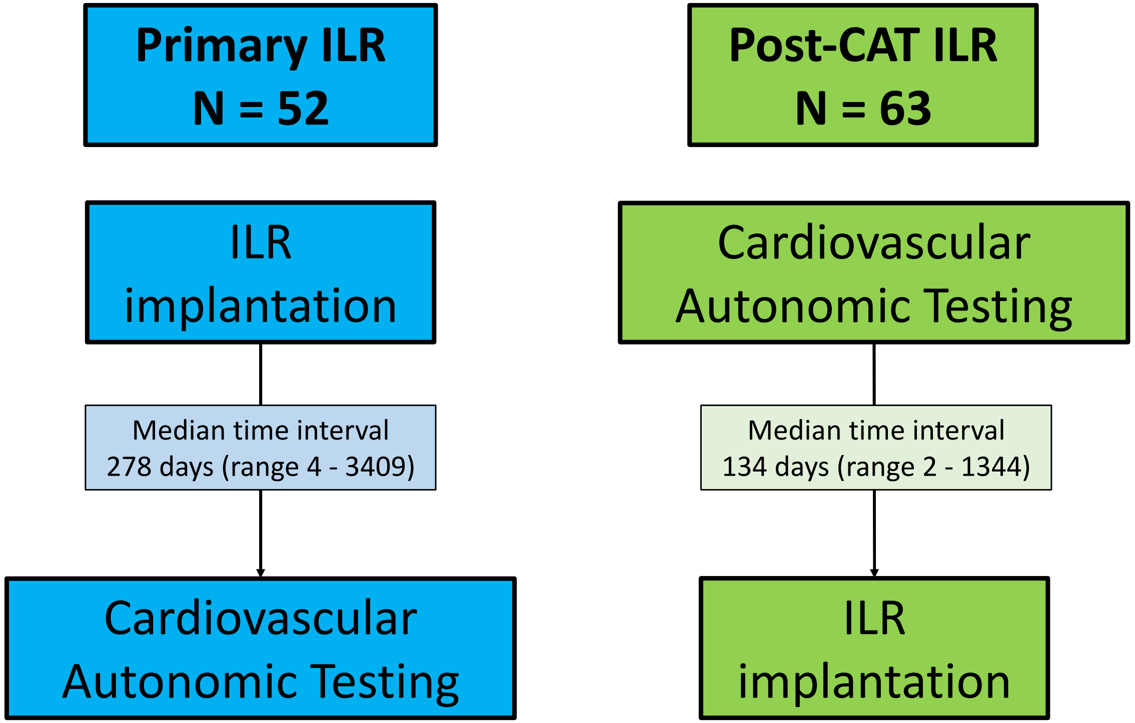

Table S1. Individual patient diagnosis by post-CAT or primary ILR strategy

Post-CAT (delayed) ILR implantation, N=63			
**Age**	**Sex**	**ILR Dx**	**CAT Dx**
74	W	NSR	VVS
79	M	NSR	NO DX
50	M	NSR	NO DX
82	W	NSR	NO DX
61	M	NSR	VVS
84	W	NSR	NO DX
75	W	AVB	OH
74	W	AVB	NO DX
56	M	AVB	VVS
72	W	AVB	VVS
71	W	AVB	NO DX
72	W	AVB	VVS
74	W	AFIB	OH
27	W	VT/VF	VVS
80	W	SSS	NO DX
63	W	NSR	OH
67	W	NSR	CSH
79	W	NSR	OH
84	W	AFIB	NO DX
71	M	AFIB	VVS
72	W	AFIB	NO DX
51	W	AFIB	NO DX
70	M	AFIB	OH
77	W	AFIB	NO DX
66	W	AFIB	NO DX
64	W	AFIB	VVS
57	W	SVT	NO DX
64	W	SVT	VVS
39	M	SVT	VVS
35	W	SVT	NO DX
57	W	SVT	NO DX
57	W	ST	VVS
27	W	NSR	VVS
18	W	NSR	VVS
29	W	NSR	NO DX
57	M	NSR	CSH
61	M	NSR	NO DX
73	W	NSR	NO DX

70	W	NSR	VVS
71	M	NSR	OH
41	W	NSR	VVS
50	M	NSR	OH
23	W	NSR	NO DX
81	W	NSR	VVS
20	W	NSR	NO DX
70	W	NSR	OH
42	M	NSR	NO DX
70	M	NSR	CSH
81	W	NSR	NO DX
48	M	NSR	NO DX
49	M	NSR	NO DX
77	W	NSR	VVS
68	W	NSR	CSH
38	W	NSR	NO DX
53	W	NSR	NO DX
58	W	NSR	OH
72	W	NSR	OH
76	M	NSR	CSH
74	W	NSR	NO DX
58	W	NSR	OH
72	W	NSR	NO DX
53	M	NSR	NO DX
74	M	NSR	NO DX
**Primary ILR implantation, N=52**			
**Age**	**Sex**	**ILR Dx**	**CAT Dx**
62	M	SSS	NO DX
79	M	SSS	CSH
58	M	SSS	NO DX
54	W	AVB	OH
65	W	AVB	VVS
77	M	AVB	OH
76	M	AVB	NO DX
77	M	NSR	CSH
65	W	NSR	VVS
63	M	AF	VVS
79	M	AF	VVS
81	M	AF	OH
50	W	AF	NO DX
74	W	VT/VF	OH
42	W	SVT	VVS

63	M	SVT	NO DX
75	W	SVT	NO DX
21	W	ST	VVS
12	M	ST	VVS
87	W	ST	CSH
22	W	NSR	VVS
27	W	NSR	NO DX
76	W	NSR	VVS
62	M	NSR	OH
54	W	NSR	NO DX
26	W	NSR	NO DX
67	W	NSR	CSH
70	W	NSR	NO DX
85	W	NSR	VVS
18	M	NSR	VVS
62	M	NSR	OH
20	M	NSR	VVS
65	W	NSR	NO DX
60	M	NSR	VVS
29	W	NSR	VVS
15	W	NSR	VVS
75	M	NSR	VVS
62	M	NSR	VVS
45	M	NSR	VVS
58	W	NSR	VVS
62	W	NSR	CSH
55	M	NSR	VVS
44	M	NSR	NO DX
36	M	NSR	VVS
29	M	NSR	NO DX
39	M	NSR	VVS
60	M	NSR	VVS
20	W	SVT	OH
56	W	NSR	VVS
47	W	NSR	OH
62	W	NSR	NO DX
56	M	NSR	OH

AF, atrial fibrillation; AVB, atrioventricular block; CAT, cardiovascular autonomic testing; CSS, carotid sinus syndrome; Dx, diagnosis; ILR, implanted loop recorder; NSR, normal sinus rhythm; OH, orthostatic hypotension; SSS, sick sinus syndrome; ST, sinus tachycardia; SVT, supraventricular tachycardia; VT/VF, ventricular tachycardia/ventricular fibrillation; VVS, vasovagal syncope.
